# The push-pull principle: an electrostatic actuator concept for low distortion acoustic transducers

**DOI:** 10.1038/s41378-022-00458-z

**Published:** 2022-11-30

**Authors:** Bert Kaiser, Hermann A. G. Schenk, Lutz Ehrig, Franziska Wall, Jorge M. Monsalve, Sergiu Langa, Michael Stolz, Anton Melnikov, Holger Conrad, David Schuffenhauer, Harald Schenk

**Affiliations:** 1 Fraunhofer Institute for Photonic Microsystems IPMS, Dresden, Germany; 2 Arioso Systems GmbH; since Sep. 1st 2022: BOSCH Sensortec GmbH, Dresden, Germany; 3 Chair of Micro and Nano Systems, Brandenburg University of Technology Cottbus-Senftenberg, Cottbus, Germany

**Keywords:** Electrical and electronic engineering, NEMS

## Abstract

Electrostatic actuators are of particular interest for microsystems (MEMS), and in particular for MEMS audio transducers for use in advanced true wireless applications. They are attractive because of their typically low electrical capacitance and because they can be fabricated from materials that are compatible with standard complementary metal-oxide semiconductor (CMOS) technology. For high audio performance and in particular low harmonic distortion (THD) the implementation of the push-pull principle provides strong benefits. With an arrangement of three electrodes in a conjunct moving configuration on a beam, we demonstrate here for the first time a balanced bending actuator incarnating the push-pull principle operating at low voltages. Our first design already exhibits a harmonic distortion as low as 1.2% at 79 dB using a signal voltage of only 6 V_p_ and a constant voltage of only ±10 V_dc_ in a standard acoustic measurement setup. Thus, exceeding our previously reported approach in all three key performance indications at the same time. We expect that our novel electrode configurations will stimulate innovative electrostatic actuator developments for a broad range of applications. In this paper we report the basic theory, the fabrication and the performance of our novel actuator design acting as an audio transducer.

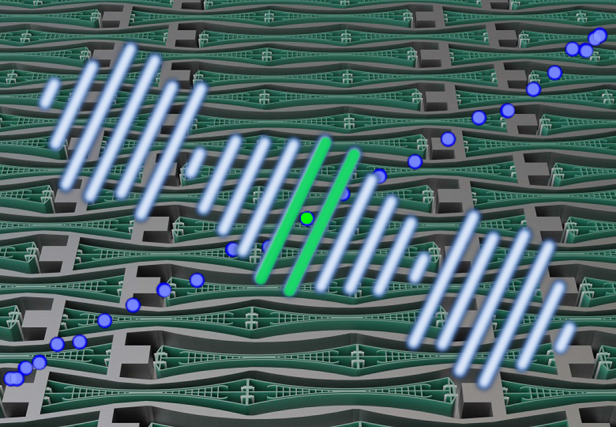

## Introduction

Highly linear MEMS μSpeaker technology is undoubtedly a current focal point of MEMS research. This interest is predominantly fueled by the desire for enhanced internet connectivity provided by true wireless headsets. And with the prospect of MEMS μSpeakers which draw only minute reactive currents at high sound pressure levels, innovative and cost-effective solutions – even for hearing aids – come within reach.

Quite impressive MEMS-based speakers have been reported in the literature. They are based on different transduction mechanisms, various materials and fabrications processes respectively.

One way of comparing the devices is their footprint, i.e. their geometric dimensions, in accordance with the attained sound pressure level in specific acoustic setups. For μSpeakers with dimensions in the sub-centimeter domain, a closed cavity approximating the ear canal (1.26 cm^3^, according to IEC 60318-4 standard) is proposed as the acoustic setup. Specifically, the harmonic distortion should be as low as possible at a specific sound pressure. Furthermore, the capacitive load of a device is a profound way to estimate the relative power consumption of the reported approaches. A capacitance of more than a few nano-Farads gives rise to reactive peak currents, which are unbearable for batteries typically used in modern true wireless headsets, at the required sound pressure levels (SPL) of 125 dB or above in the frequency domain of human speech.

MEMS μSpeaker approaches rely on the piezoelectric transduction mechanism^1–8^, the electrostatic transduction mechanism^9–15^, the magnetic transduction mechanism^16–20^ and the thermoacoustic transduction mechanism^21,22^. A thorough review paper about micro speakers was recently published by Wang et al.^23^. A few publications shall be highlighted in the following.

Recently Wang et al. described a MEMS μSpeaker based on thin lead zirconium titanate ceramic^8^. The device measures 6.7 × 6.7 mm^2^ and has a circular membrane of about 4.2 mm diameter with a deflection direction, which is out-of-plane. The reported SPL reached 119 dB at a distance of 10 mm and for a frequency of about 9 kHz. This translates to approximately 124 dB in a 1.26 cm^3^ closed cavity. An estimation from the graph reveals a value of 64 dB at a referential frequency of 500 Hz with the same assumed cavity. No harmonic distortion values are reported. Taking the given geometric dimensions, a capacitive load of at least 1.6 μF is estimated for the active area (4 mm diameter, lead zirconium titanate permittivity *ϵ*_r_ = 300, layer thickness 5 μm).

A push-pull configuration with static electrodes was reported by Zhou et al. for a speaker based on a graphene diaphragm^11^. The speaker had two static electrodes and the diaphragm acted as a shuttle which translated the Coulomb forces into sound pressure waves. The drive used was a 100 V_dc_ bias and a 10 V_p_ signal while at the same time very low power consumption, below 1 μW, and very high power efficiency were achieved. No absolute values were reported for sound pressure or harmonic distortion.

Kim et al. reported on implementing the push-pull principle in a μSpeaker^10^. Sound pressure levels of 113.4 dB and 98.8 dB were attained at 1 cm distance using two and one electrodes respectively. This translates to about 121 dB and 96 dB in a closed cavity of 1.26 cm^3^ volume. No values for harmonic distortion or electrical capacitance were reported.

Sugimoto et al.^24^ reported an acoustic transducer based on two dielectric elastomer films which specifically took advantage of the push-pull principle to suppress harmonic distortion. A decline in harmonic distortion of 10 dB (factor ≈ 3) was described for push-pull driving over one-side driving.

Similarly Hsin-Yuan Chiang and Yu-Hsi Huang reported the experimental modeling and application of push-pull electrostatic speakers^25^. Their devices are not in the micro-sized speaker domain because they incorporate a circular diaphragm of at least 60 mm in diameter. Specifically, the authors claim an outstanding sound quality for their push-pull speakers.

In our own work^26^ we reported on a MEMS μSpeaker based on electrostatic bending actuators deflecting laterally in-plane. A sound pressure of 69 dB in a 1.26 cm^3^ cavity was reported with a harmonic distortion below 4.4% at a driving voltage of 40 V_dc_ plus 5 V_p_ (pull-in voltage *U*_pi_ = 49 V). The device’s active area measured 9.3 mm^2^ and it exhibited an electric capacitance of 65 pF.

This literature overview reveals several works addressed to MEMS μSpeaker. Some of them implement configurations based on the push-pull concept. These approaches are assumed to be either large and/or require quite high driving voltages because of the use of static electrodes. The electrostatic push-pull actuator concept offers strong benefits^27^. Especially highest linearity achievable regarding voltage-deflection relation is a dominant quality of such actuators. Nevertheless, the implementation of an electrostatic push-pull actuator configuration remained challenging due to the arrangement of the electrodes and their respective relative movement with respect to each other. Likewise, this affects their voltage-deflection relation as well as the device design in which they are implemented in. Up to now, the general drawback of electrostatic actuators has been the necessity to operate them at comparably high voltages. This applies in particular to electrostatic actuators in a push-pull configuration, which in case of μSpeakers is required to achieve low harmonic distortion. Ultimately, the high voltage requirement is linked to the classical stator-shuttle electrode configuration and the limits posed by the pull-in phenomenon - a singularity.

Singularities attract many engineers’ interest. In the case of electrostatically driven MEMS μSpeakers, such critical behavior is encountered in extremely miniaturized, high fidelity audio reproduction systems that are supposed to operate at high sound pressure levels. Typical MEMS μSpeaker designs use an elastic force to balance an electrostatic force. These two forces are quite different in nature. This asymmetry creates a singularity, called pull-in, when the input signal generates an electrostatic force strong enough to annihilate the relevant effective stiffness. For state-of-the-art μSpeaker designs, the demand for high audio reproduction quality in portable applications implies the need to operate them close to the pull-in. Only close to the pull-in is the slope of the deflection curve, i.e. the sensitivity of the transducer, large enough to produce high sound pressure levels at signal voltages sufficiently low for portable applications. Additionally, only when the μSpeaker is driven at low signal amplitudes the total harmonic distortion (THD) is acceptably low. The THD is defined as

1 $$THD=100\sqrt{1-\frac{{K}_{1}^{2}}{\sum {K}_{n}^{2}}},$$

with *K*_*n*_ being the harmonic components of the sounds generated.

For practical use, operating an electrostatic transducer close to pull-in is not desirable. Unless special protective measures have been taken, such a modus operandi is prone to catastrophic failure, caused, e.g., by minute mechanical shocks.

**Fig. 1:**
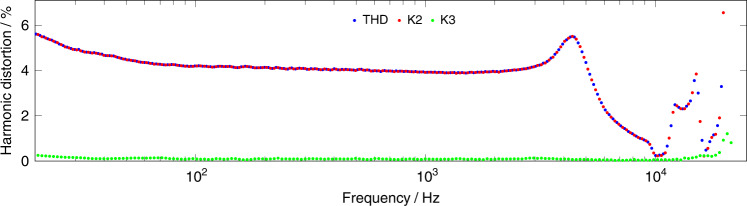
μSpeaker with asymmetric actuator. Acoustically measured THD for a μSpeaker employing solely-pull bending actuators. The THD is dominated by the 2^nd^ harmonic K2 due to the quadratic relation between signal voltage and displacement^26^

The other relevant implication of the electroelastic asymmetry is the generation of a pronounced second harmonic distortion (K2). That K2 tends to keep the THD well above an acceptable threshold of about 1% (Fig. 1) for μSpeaker employing solely-pull bending actuators. Compensating for such a deficiency by means of advanced signal processing is not desirable in portable devices that work off a tight battery budget. Instead the approach is to limit the share of K2 on the THD and go down to the third harmonic distortion (K3) fraction, which is significantly lower.

The question arises of how to design an electrostatic MEMS transducer, suitable for broad frequency range operation which have low harmonic distortion and do not need to be operated close to the pull-in singularity. The classical answer to this question is to use a push-pull configuration, placing a shuttle electrode between two stator electrodes^28^. However, due to the required large stator distance, such push-pull designs, even when implemented as MEMS, operate at high voltages that are impractical for portable applications, and in particular for in-ear use. In previous papers^26,29^, we reported an asymmetric design that solves the large voltage issue.

Here we show for the first time that the stator-shuttle configuration can in fact be replaced by a design that integrates all three electrodes into the moving parts of an electrostatic bending actuator. This means the electrode spacings or “gaps” can be kept tight, allowing voltages suitable for small portable devices and at the same time achieving a much-improved linearity of the resulting audio reproduction without sacrificing the maximum attainable sound pressure levels. In this work we describe such a concept for an electrostatic actuator based on MEMS technology. First, we introduce the transducer design and secondly, we introduce the bending actuators principle, theoretical description and fabrication. As a proof, we demonstrate a μSpeaker showing low THD levels as well as low voltage requirements while at the same time maintaining sufficient SPL levels.

## Transducer design

**Fig. 2:**
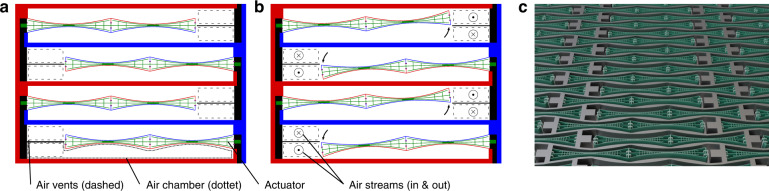
μSpeaker employing push-pull bending actuator. Schematic top view and 3D visualization; **a** schematic top view of μSpeaker concept with one pair of push-pull bending actuators in initial state, w/o voltages; voltage distribution is depicted by different colors (e.g. red/blue ± *u*_dc_. Green *u*_ac_) **b** same view as in (**a**) but in deflected state exhibiting actuators bending in an odd mode relative to each other with constant voltages as well as signal voltage applied (at a time of *u*_ac_ being at its amplitude value). **c** Schematic 3D visualization of actuator arrangement in initial state, artistic colorization

The core component of the low distortion μSpeakers presented in this work is the balanced bending actuator based on the push-pull principle. The MEMS μSpeaker concept is based on a stack of wafers pile up to create a device layer sandwiched between a bottom and a cover wafer. A plurality of actuators and air chambers are preferably arranged densely and actuated simultaneously for sound reproduction. However, the push-pull concept has several ramifications for the design of the acoustic transducer. The bending actuator is clamped on one side only. One-side clamping is favored due to linearity concerns over the Duffing effect created by stress stiffening in two-side clamped beam designs. The push-pull actuators operate in single and individual air chambers. These air chambers are defined by the actuators themselves and additional sidewalls between each of the actuators. Fig. 2a shows the layout concept. The actuators are arranged in rows and columns. In each column the actuators alternate with respect to their clamped side. If actuated, the push-pull actuators bend towards each other or away from each other. Thus, two neighboring actuators form a pair, canceling out the resulting inertial forces. Close to its free end, where the maximum deflection and air displacement are achieved, each actuator has its own pair of air vents. The actuator tip is separated from the opposing silicon surface by a small gap. Like the clearance, this gap is small enough not to cause acoustic leakage and ensures the movability of the actuators. It should be noted that the neighboring actuators are shifted by about half a cell pitch (Fig. 2a), to allow for denser packing and higher area utilization. The sidewalls between the actuators transfer the electrical potential between the alternating clamping sides of the beams within the chip. Technological cleanroom efforts, as well as wiring expenses, are greatly reduced because crossing potentials or external bridges or wires are avoided. While this is true for both constant voltages, which can be distributed within the device layer in the way described, it is necessary to integrate the bottom wafer as an electrical distribution layer for the audio signal. This is done by introducing through-silicon vias electrically connecting the device layer and the bottom wafer specifically at the middle electrode. With these means of potential distribution, no external wiring is necessary except one general connection for each potential. Additionally, it allows for the odd mode movement of the neighboring actuators and also means the air chambers are free of an electrostatic field. This is highly desired in order not to generate any electrostatic air filtering effects. When a signal voltage is applied, the actuators bend towards or away from each other depending on the sign of the signal (Fig. 2b).

The description given in the previous paragraphs mainly focuses on a lateral view of the actuator and chip design, i.e. the layout composition parallel to the MEMS surface. The actual device is then an extrusion of that layout into the chip’s volume, unleashing the uniqueness of the underlying μSpeaker concept.

**Fig. 3:**
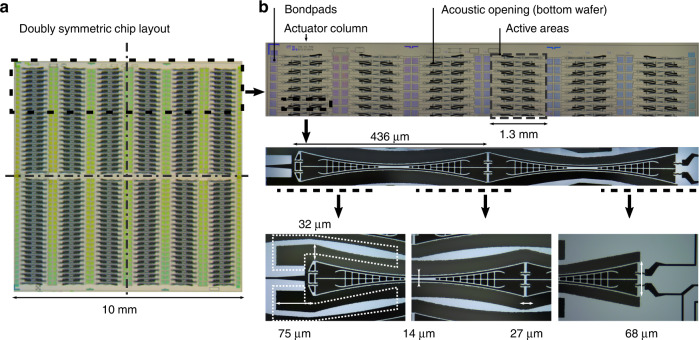
Fabricated MEMS μSpeaker device. **a** Device layer top view revealing actuator arrangement. **b** Partial view of top view and views of a push-pull actuator with geometric dimensions

Figure 3a shows the top view of the 75 μm thick device layer of a fabricated MEMS speaker. The whole chip comprises 276 actuator pairs arranged in two groups. Both groups are of the same size and design, and actually mirror each other with respect to the center of the chip (horizontal dashed-dotted line). These groups are defined by the wiring scheme within the device plane and are individually addressable. The reason for having those two groups is purely down to testing capabilities.

Each actuator has a length of 872 μm and maximum and minimum widths of about 68 μm and 14 μm respectively. A vertical pull-in between actuator and cover/bottom wafer for the maximum driving voltage of 36 V is safely avoided. Additional holes have been placed within the center electrode. These holes do not affect the electromechanical behavior but do lower the mass of the actuator. The resonance frequency was calculated as about 5 kHz for the first mode, which is the desired in-plane swiping movement.

The actuators are placed pairwise in an active area (pitch) of 200 μm (vertically, rows) by 1646 μm (horizontally, columns). The vertical pitch is designed in such a way that the maximum deflection in resonance still gives enough room for the air to be exerted from the chambers through the vents. Specifically, a maximum quasistatic tip deflection of the actuators of about 8 μm peak to peak with a excitation in resonance and a remaining distance each of around factor two was assumed. This leads to a distance of 32 μm between actuator edge at the tip and closest wall (Fig. 3b). The air vents were designed to about 40 × 260 μm in width and length respectively. The relatively large air vents overlap the sidewalls partially, making it possible that air is exerted from two neighboring actuators (see dotted indication in Fig. 3b). The chip design incorporates two individual bond pads (170 × 70 μm^2^) for the constant voltages in each group of actuators, i.e. four in total. The third potential is to be contacted via the bottom wafer as a backside contact or via individual bond pads at each actuator. The upper frame of the MEMS chip shows additional structures relevant for technological process monitoring purposes only. The whole chip measures 10 × 10 mm^2^ including the chip frame and has a total thickness of about 1.1 mm. The device layer has a thickness of 75 μm and the bottom and cover wafers are 625 μm and 400 μm thick respectively. The separation between the device layer and the bottom layer is maintained by the 1 μm buried oxide layer. The cover wafer and device wafer showed a separation (clearance) of the exact same size.

## Theory

### Push-pull-concept

**Fig. 4:**
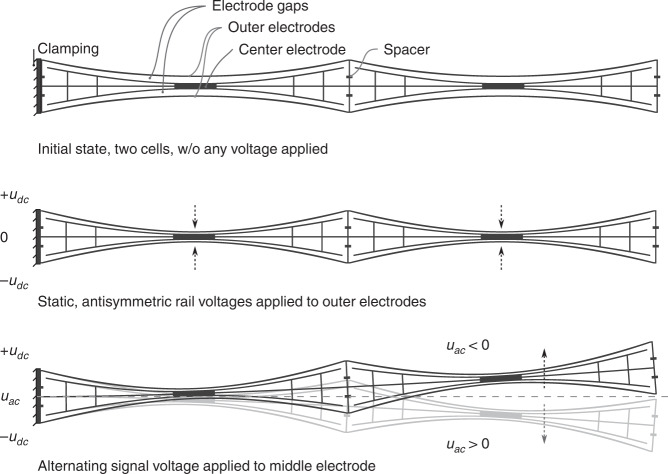
Push-pull bending actuator in μSpeaker. Schematic top view; symmetrical three-electrode arrangement forming a laterally deflecting beam

The push-pull concept for electrostatic bending actuators is shown in Fig. 4. It shows three independently addressable electrodes placed next to each other in close proximity. In fact, the distance is defined by the two electrostatic gaps. These gaps can be kept small in order to allow for small driving voltages. The minimum width is most likely defined by technological constraints. All three electrodes are mechanically linked together and form a group of simultaneously moving mechanical entities. The overall appearance of the group is like a beam because of the elongated geometric dimensions of the electrodes. The beam and group of electrodes are symmetrical with respect to their centered longitudinal axis. This symmetrical layout of the beam allows the non-linear electrostatic forces in the gaps at both sides of the neutral fiber to be counteracted. The actual shape of the actuator is less similar to a parallel plate and more likely to be recognized as suspension bridge arrangement. The reason for this is its mechanical susceptibility to bending in combination with the general desire to have the lowest area consumption possible.

Bending of the beam-like actuator is established by incorporating the nanoscopic electrostatic drive (NED) principle^29^. Coulomb forces in the gap are transferred into uniaxial stress by the shape of the electrodes, thus bending the actuator. Constant voltages *u*_dc_ of the same amplitude (with respect to the middle electrode) but with opposing signs are applied to the actuator’s outer electrodes. In this state, no actual deflection in a lateral sense takes place. The audio signal *u*_ac_ is then applied to the middle electrode, thus unbalancing the forces on both sides of the actuator. In this state the actuator is actively bending in one of the two directions depending on the sign and amplitude of the audio signal. The direction is determined by which gap exhibits the greater voltage difference of *u*_dc_ + *u*_ac_. In other words, one gap has electrostatic forces pulling more strongly in its direction - “pull” - and the opposing gap has electrostatic forces pulling less strongly in the opposing direction - “push”. Interestingly, the actuator behaves like a push-pull system without pushing or repelling forces being present. Consequently, a linearized deflection is generated.

The design process can be understood as follows. The variable of merit is the achievable deflection for a given set of driving voltages. Respecting technological capabilities, which define the minimum electrode thicknesses as well as the minimum gap distances, the deflection will be maximized by using the thinnest electrodes and the smallest gaps possible. Assuming a fixed and already minimized stiffness, the mass of the actuators, i.e. their cross-sectional area, should be minimized for a resonance frequency suitable for in-ear applications. Consequently, the aforementioned suspension-like structure is superior to a reciprocal dome-like shape, due to less confined area by the actuators contour. The actual design details were determined using finite element analysis in a parameter sweep, in order to account for all the rather complex design rules. More details on the finite element analysis are given in section “Material and methods”.

Some basic equations explaining the essence of the acoustic performance of our push-pull actuators are discussed in the following section.

### Theoretical description

**Fig. 5:**
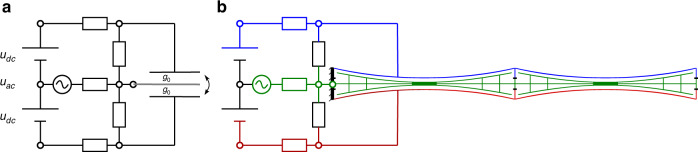
Representation of moving push-pull electrode arrangement. **a** electrical circuit representation with moving shuttle between two static electrodes **b** circuit attached to schematic actuator

The classical electrostatic push-pull principle is depicted in Fig. 5a. It comprises a shuttle electrode that moves between two stator electrodes which are held at constant voltages in a two-loop circuit topology. When used in audio transducers, a classical electrostatic push-pull operation requires the use of very high drive voltages, typically of the order of 1000 V^30^. Combining the push-pull principle with the NED concept as outlined in the preceding section allows, in contrast, for a low voltage operation, as is required for true wireless in-ear applications (Fig. 5b).

The most basic, though very effective, method modeling a NED-based push-pull actuator, is introducing a mechanical lever factor *θ* into the classical equation for an electrostatic push-pull actuator. In its dimensionless form this equation reads

2 $$\frac{{{d}}^{2}a(\tau )}{{d}{\tau }^{2}}+\frac{1}{Q}\frac{{d}a(\tau )}{{d}\tau }+k\,a(\tau )=\theta \left[{\left(\frac{{u}_{dc}+{u}_{s}(\tau )}{1-a(\tau )}\right)}^{2}-{\left(\frac{{u}_{dc}-{u}_{s}(\tau )}{1+a(\tau )}\right)}^{2}\right].$$

Here *a*(*τ*) is the dimensionless stroke of the actuator^28^ normalized to the initial electrode spacing *g*_0_. The dimensionless quantities *τ*, Q, *u*_*dc*_ and *u*_*s*_(*τ*) are the dimensionless time, the quality factor, the dimensionless constant voltage and the dimensionless signal voltage respectively. The dimensionless stiffness *k* is typically normalized to unity. We keep it here to facilitate the interpretation of the formulae below. While the definition of the stroke is entirely obvious in the case of a rigid-plate-like shuttle, its meaning is less clear in the case of an elastic electrode that changes its shape under the influence of the Coulomb force. However, this dynamic variable can be identified, in a very well-defined sense, with the maximum deflection of a Coulomb-actuated Euler-Bernoulli beam, as used here^31–33^.

The deflection characteristics in the case of a constant signal voltage are depicted in Fig. 8b. The domain of stable device operation extends between the two bifurcation points where the slope of the deflection curve reaches infinity (pull-in). The slope of the deflection curve at the origin can be tuned by the value of the constant voltage.

As can be expected from the shape of this curve, the push-pull principle allows for device operation with the signal voltage oscillating about zero and with low harmonic distortion. This is in sharp contrast to the asymmetric electrostatic drive^26,31^, where a working point close to the pull-in must be selected in order to minimize harmonic distortions. To analyze this claim in greater depth, we observe that according to Eq. (2) the direction of the stroke is reversed upon changing the sign of the signal voltage,

3 $${u}_{s}(\tau )\to -{u}_{s}(\tau )\quad \Rightarrow \quad a(\tau )\to -a(\tau ).$$

The consequences of this inversion symmetry can conveniently be understood by a deformation (homotopy) argument. To this end, we select a signal voltage oscillating around zero,

4 $${u}_{s}(\tau )={u}_{ac}\cos (\omega \tau ),$$

and expand the stroke in terms of the signal amplitude,

5 $$a(\tau )=\mathop{\sum }\limits_{n=0}^{\infty }{{\mathfrak{a}}}_{n}(\tau ){u}_{ac}^{n}.$$

From the inversion symmetry we can immediately infer that all coefficient functions with even indices vanish,

6 $${a}_{2n}(\tau )=0\quad \quad n\in {{{\bf{N}}}}.$$

Inserting Eq. (5) into Eq. (2), expanding the non-linear terms in *u*_*ac*_ and collecting all terms of a given power in *u*_*ac*_, we find that the coefficient functions ${{\mathfrak{a}}}_{n}(\tau )$ satisfy the following deformation equations:

7 $$\frac{{{d}}^{2}}{{d}{\tau }^{2}}\,{{\mathfrak{a}}}_{n}(\tau )+\frac{1}{Q}\frac{{d}}{{d}\tau }\,{{\mathfrak{a}}}_{n}(\tau )+(k-4{u}_{dc}^{2})\,{{\mathfrak{a}}}_{n}(\tau )={{\mathfrak{f}}}_{n}(\tau ).$$

The driving forces ${{\mathfrak{f}}}_{2n}(\tau )$ with even indices vanish, as expected,

8 $${{\mathfrak{f}}}_{2n}(\tau )=0\,\quad n\in {{{\bf{N}}}}.$$

The driving forces for odd indices are computed to be,

9 $$\begin{array}{llll}{{\mathfrak{f}}}_{1}(\tau )\,=\,4{u}_{dc}\cos (\omega \tau ),\\ {{\mathfrak{f}}}_{3}(\tau )\,=\,4{\cos }^{2}(\omega \tau ){{\mathfrak{a}}}_{1}(\tau )+12{u}_{dc}\cos (\omega \tau ){{\mathfrak{a}}}_{1}{(\tau )}^{2}+8{u}_{dc}^{2}{{\mathfrak{a}}}_{1}{(\tau )}^{3},\\ {{\mathfrak{f}}}_{5}(\tau )\,=\,8{\cos }^{2}(\omega \tau ){{\mathfrak{a}}}_{1}{(\tau )}^{3}+20{u}_{dc}\cos (\omega \tau ){{\mathfrak{a}}}_{1}{(\tau )}^{4}+12{u}_{dc}^{2}{{\mathfrak{a}}}_{1}{(\tau )}^{5}\\ \qquad \quad\,\,+\,24{u}_{dc}^{2}{{\mathfrak{a}}}_{1}{(\tau )}^{2}{{\mathfrak{a}}}_{3}(\tau )+24{u}_{dc}\cos (\omega \tau ){{\mathfrak{a}}}_{1}(\tau ){{\mathfrak{a}}}_{3}(\tau )\\ \qquad \quad\,\,+\,4{\cos }^{2}(\omega \tau ){{\mathfrak{a}}}_{3}(\tau ),\\ \qquad \qquad\,\,{{{\rm{etc.}}}}.\end{array}$$

This hierarchy of equations can be solved recursively, beginning at ${{\mathfrak{a}}}_{1}(\tau )$ and progressing from one index to the next, higher index. Note that the force term at any level of the recursion only depends on coefficient functions already known from previous steps. At each step only a linear equation must be solved, which can be readily done by purely algebraic means, based on the usual Laplace transformation argument. Note in particular that ${{\mathfrak{a}}}_{1}(\tau )$ only contains the first harmonic. Using this result we are able to compute the explicit form of the driving force ${{\mathfrak{f}}}_{3}(\tau )$. Reducing the involved trigonometric polynomials to simple harmonics, we find that this driving force has only odd harmonics components up to 3^rd^ order. This implies that ${{\mathfrak{a}}}_{3}(\tau )$ also only contains odd harmonics up to 3^rd^ order. The argument can be repeated for any higher order, proving the claim that a perfectly balanced push-pull actuator entirely suppresses all even harmonic distortions, and in particular the second harmonic distortion (K2). Therefore, a perfectly balanced push-pull actuator only produces odd harmonics, predominantly the third harmonic (K3), which should lead to a substantially better performance compared to asymmetric driving schemes^28^.

The procedure outlined above can easily be used to actually compute the THD, as a power series in *u*_*ac*_. The first three harmonic components *K*_*n*_ of the THD, are given for a perfectly balanced actuator up to order $O{[{u}_{ac}]}^{5}$ and for *θ* = 1 by

10 $$\begin{array}{rcl}{K}_{1}&=&4{u}_{dc}{u}_{ac}\left\vert \frac{Q}{kQ+\omega (i-Q\omega )-4Q{u}_{dc}^{2}}+\frac{{Q}^{2}\left((kQ+\omega (i-Q\omega ))(3kQ+\omega (i+3Q\omega ))\right)+4Q(3kQ+\omega (i-3Q\omega ))}{(kQ+\omega (i-Q\omega )-4Q{u}_{dc}^{2})(kQ+\omega (i+Q\omega )-4Q{u}_{dc}^{2})}{u}_{ac}^{2}\right\vert ,\\ {K}_{2}&=&0,\\ {K}_{3}&=&4{u}_{dc}{u}_{ac}^{3}\left\vert \frac{{Q}^{2}\left(kQ+\omega (i-Q\omega )\right.(kQ+\omega (i-Q\omega )-4Q{u}_{dc}^{2})}{(kQ+3\omega (i-3Q\omega )-4Q{u}_{dc}^{2}){(kQ+\omega (i-Q\omega )-4Q{u}_{dc}^{2})}^{3}}\right\vert .\end{array}$$

The results for non-trivial *θ* are obtained by scaling all voltages by a factor of $\sqrt{\theta }$. These formulae nicely fit numerical simulations. More importantly, the efficient algorithm sketched here serves well as a solid base for analyzing the impact of imperfect balancing. As is well known from the theory of Volterra series^34–36^, the radius of convergence of Eq. (5) extends to the point where the magnitude of the signal amplitude causes a dynamic instability. This situation is reached when the frequency response curve develops a Duffing-type multivaluedness^31,37^. We will not dwell on the details of such analysis here, but we share the results in additional publications^32,38^. For comparison with our experimental findings, we also need to add a suitable acoustic load. This is done in the network model used below.

## Fabrication

**Fig. 6:**
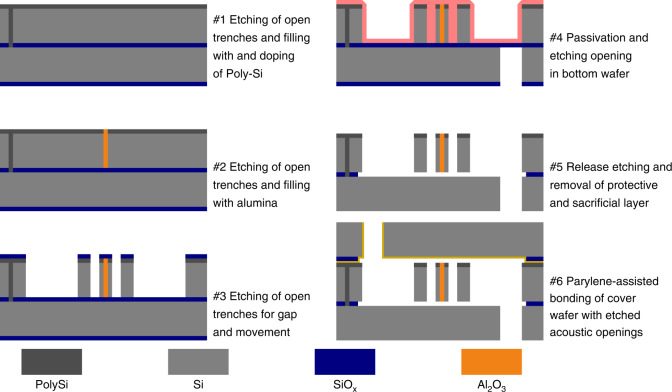
µSpeaker fabrication process flow. Schematic representation (not to scale) of main process steps in technology

The linear bending actuators were fabricated on a single chip design. The basic wafer material was bonded silicon on insulator wafers with a phosphor dopant concentration of 10^18^ cm^−3^ for the device layer, resulting in a specific electrical resistance of 0.01–0.025 Ω cm. The main process step for the bulk micro-machining was deep reactive ion etching. Layer deposition for trench filling as well as cover wafer bonding complete the technological processes (Fig. 6). A 200 mm diameter wafer had 197 devices arranged on it. Details of the technology and processes used are supplied in the following.

The linear bending actuators were fabricated starting with bare bonded silicon on an insulator wafer. The first step was the creation of the through-silicon vias. The about 2.4 μm wide holes were etched by deep reactive ion etching (DRIE), subsequently followed by an etch step to remove the buried oxide otherwise acting as an electrical insulator. The holes or trenches were afterwards filled with polysilicon using low pressure chemical vapor deposition. The deposition was split into two steps of about 1000 nm deposition layer thickness. The intermediate doping of the polysilicon was achieved by applying an atmosphere of phosphoroxychloride (POCl_3_) at 950 °C for about 20 min. This sequence was repeated for the second layer of polysilicon. Another set of trenches was etched, again utilizing DRIE and subsequently filled with alumina using atomic layer deposition (1800 nm, 300 °C). The alumina-filled trenches formed the spacers between the electrodes in the linear bending actuator. Finally, device layer etching was completed by etching open trenches again to build the actuator shape, sidewalls and air chambers. Note that this etch step is crucial for the actuators to work properly. Etch insufficiencies in this step are likely inducing two main failure causes. First, conduction paths may remain between electrodes inducing a short cut, thus inhibiting the creation of an electrical field and the genesis of electrostatic forces respectively. Second, electrodes are not etched homogeneously into the depth, i.e. exhibiting a thinning of the electrode with regard to the depth. Following, such thinned electrodes and actuators respectively may not sustain voltages and forces as high as their neighboring actuators causing them to fail early likely because of pull-in. Subsequently openings in the handle wafer were DRI-etched from the backside through 650 μm of silicon to create the air inlets and outlets in the bottom wafer. Finally, part of the oxide between device and handle layer was removed by exposing the wafer to several cycles of hydrogen flouride (HF) which released the actuators and ensures movement capabilities. In parallel, the cover wafer was prepared by etching a silicon step of 1 μm and subsequently etching the inlets and outlets through 400 μm of silicon, both by using DRIE. The wafer stack was completed by gluing the cover wafer using parylene to assist wafer bonding^39^. A layer of 500 nm of parylene was deposited on the cover wafer. Afterwards the device wafer is aligned and clamped to the cover wafer and bonded at 300 °C and 2000 mbar of uniform pressure. The parylene will remain as baked glue holding both wafers tightly together. Subsequently, the wafer is separated into individual devices using stealth dicing. An infrared laser weakens the silicon between the chips during multiple passes with different focus depths. Finally, actual cleavage is achieved by expansion of the carrier foil introducing breakage at the respective weakened paths within the silicon.

## Measurement setup and results

**Fig. 7:**
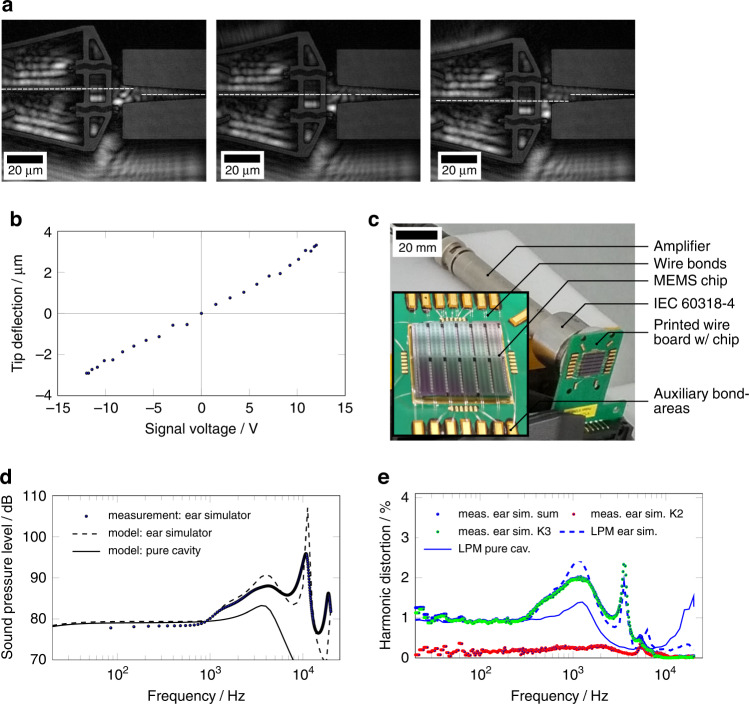
MEMS specimen setup and characterization. **a** deflection states of actuator for both deflection directions, dashed lines indicate deflection. **b** in-plane deflection measurement data obtained with edge tracking (sinusoidal excitation at 50 Hz, ± 12 V_dc_) **c** MEMS device on carrier board including wire bonds. **d** sound pressure level measurement data ( ±10 V_dc_, ±6 V_p_). **e** THD measurement data ( ±10 V_dc_, ±6 V_p_), THD and K3 measured data overlap, while K2 fraction remains very low

To optically access the bending actuators, the devices were examined before the cover wafer was bonded on top. The characterization of the linear actuator deflection was carried out using a digital holographic microscope, see Fig. 7a. The edge tracking function of the associated software allowed resolution down to 163 nm. The electrical contact was established by tungsten needles. These were placed directly onto the surfaces for all three voltages (two constant voltages ±10 V_dc_ and audio signal 6 V_p_).

The optically recorded deflection characteristics in Fig. 7b clearly show the targeted inversion symmetry required in Eq. (3) to suppress the second harmonic distortion K2. This also aligns with the deflection spectra of the same type of actuator measured at its tip showing the second harmonic well below the third harmonic^40^. These measurements were taken by tracking the actuator’s tip in both deflection directions as shown in Fig. 7a. The optical measurements reveal a fairly linear deflection up to amplitudes of ±3.1 μm (±12 V_dc_, ±12 V_p_). Following the linear relations at the actuator the peak-to-peak deflection available for audio reproduction was 2.6 μm (±10 V_dc_, ±6 V_p_).

For acoustic measurements, the chips were glued to a printed wire board (Fig. 7d), using conductive glue, thus providing electrical contact to the bottom wafer. Wire bonds (20 μm diameter, aluminum wires) were attached to the bond pads on the bare silicon surface. Before the acoustic test, we ran a simple electrical test using voltage sweeps up to the designed maximums. This allowed us to identify groups of actuators that were short-circuited or suffered from pull-in at low voltages. Subsequently, such groups of actors were separated during the test. The reasons for such problems lie in the technology, which will be further optimized in the future to increase yield. Finally, only 60 actuator pairs out of 276 (total active chip area 19.8 mm^2^) were used in the acoustic measurements due to electrical yield.

The acoustic performance was characterized by measuring the harmonic distortion and the sound pressure level. The ear simulator (IEC 60318-4) was placed at the backside (bottom wafer openings) of the MEMS μSpeaker device. The front side was left open to the environment. A small amount of circuitry was used to create both constant voltages (±10 V_dc_) with a virtual ground. A voltage amplifier (Krohn-Hite Model 7602M) was used to amplify the driving signal of the audio analyzer (NTI Flexus 100). The main signal pattern was a sinusoidal excitation (6 V_p_) while sweeping the frequency.

The sound pressure level, as displayed in Fig. 7d, shows an almost constant plateau of 79 dB (logarithmic reference pressure is 20 μPa, ±10 V_dc_ and 6 V_p_) for frequencies up to 1 kHz, revealing the quasistatic transduction behavior of the linear bending actuators. No significant drop in SPL is observed below 30 Hz. The referential sound pressure at 500 Hz was ascertained as 78.4 dB. At this frequency, no influence from either dynamical effects or the ear simulator is assumed. Between 1 kHz and 7 kHz, an overall increase in SPL, as well as a peak of 88 dB at an frequency of about 4.2 kHz, can be identified. The peak is clearly related to the first mode bending resonance of the actuators, as expected from electromechanical measurements. The overall increase as well as the steep increase in SPL for frequencies above 7 kHz, is attributed to the resonance of the ear simulator. This resonance leads to an absolute peak in the SPL of more than 101 dB at a frequency of about 9.1 kHz. This peak is not related to the transducer and should be expected when using the ear simulator according to the standard IEC 60318-4.

The THD, displayed in Fig. 7e, shows a constant level of never more than 1.26% for frequencies between 20 Hz and 400 Hz, including a constant plateau at around 1.0%. At frequencies above 400 Hz, we see the consequences of a dominant K3 fraction in the harmonics, i.e. a increasing THD at a frequency that is one third of that at which the SPL increases. The THD reaches a peak value of 2% at a frequency of 1.4 kHz, matching the third harmonic of the aforementioned actuator resonance. Beyond 10 kHz the ear simulator is not specified and contributes to distortion significantly. In accordance with the theory presented above, the K2 component does not dominate the THD. Instead, the THD is dominated by the third harmonic distortion K3.

The 60 actuator pairs were measured as having an approximate electrical capacitance of 260 pF using an impedance spectrometer (Keysight E4990A). The total chip capacitance includes some parasitic capacitance originating from stray fields within the chip as well as, e.g., the bond pads. The sensitivity may be calculated assuming a 40% efficiency of the driving circuitry for the pure reactive load. A value of 97.4 dB/mW is achieved at 500 Hz, assuming 1 mW in an IEC 60318-4 ear simulator.

## Discussion

**Fig. 8:**
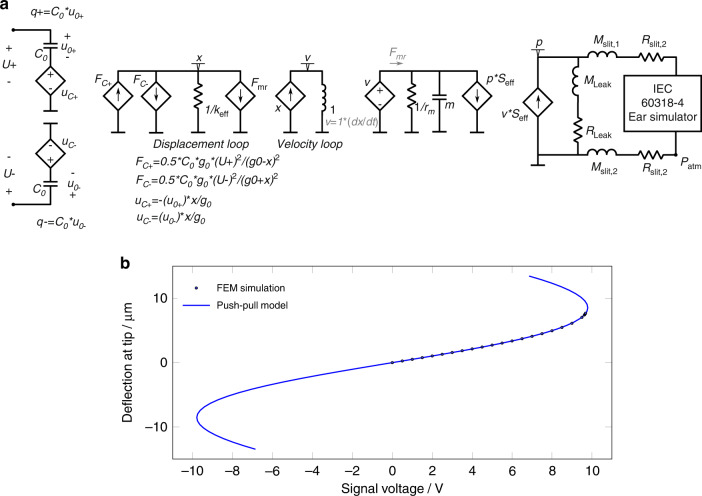
Analytical modelling of µSpeaker. **a** Large-signal equivalent circuit model of the push-pull electrostatic actuator, using the topology proposed by Monsalve et al.^33^. **b** Comparison of the fitted coefficients with the results of the finite-element simulation for 24 V_dc_

A novel, electrostatic push-pull bending actuator for low-voltage operation has been theoretically described, fabricated and characterized. Our new actuator shows a maximum deflection amplitude of 3.1 μm for a driving voltage of ±12 V_dc_ and 12 V_p_. The deflection amplitude exceeds previously reported quasistatic deflection values for electrostatic bending actuators and is normalized to the driving voltage, by a factor of 7.2 (1.5 μm at voltages of 40 V_dc_ and 5 V_p_^26^). This rise is partially attributed to the specific suspension bridge like design of the electrodes. A deviation from a linear behavior may be identified for higher voltages, visible as an increasing slope. This has to be expected due to increased effect of electrostatic and mechanical nonlinearities (ref. Fig. 8b). We demonstrated that our implementation of the push-pull principle yields a deflection, that is both, linear and symmetrical in either direction.

By implementing our novel push-pull actuators in a MEMS μSpeaker we demonstrated in particular the viability of a commercially highly attractive application. Our first balanced μSpeaker design exhibited excellent audio reproduction over a broad frequency range of more than nine octaves (10 Hz to 6.3 kHz) with a THD below 1.2%. A comparison of the measurements shown in Fig. 1 (our previous work) and in Fig. 7e shows both a significant diminution of the THD and, particularly, the reduction of the K2 component. In other words, the reduction of the THD is attributed to the diminishing K2 component following the symmetry in deflection behavior of the actuator. No signal preconditioning was required to achieve this linearity, giving rise to the argument of a possibly very efficient drive. Additionally this is supported by the electrical capacitance as low as 260 pF keeping potential reactive currents for driving low.

The chip size is 10 × 10 mm^2^. Calculation of the area-specific sound pressure level gives a value of about 53.8 dB/mm^2^. This is 4.2 dB above our previously reported value and must be highlighted in combination with the significantly reduced harmonic distortion. This is partly achieved thanks to the denser packing of the actuators within the device layer. In the specific case of our presented device one may consider all actuator pairs (276 instead of 60) being active. Theoretically, an increase in SPL of about 13 dB could be anticipated. Note that this would be the case without increasing neither driving voltages nor the THD. With ongoing development of the fabrication technology it should be possible to achieve these improvements.

The models presented in comparison to the measured values show a good approximation. Deviations are attributed to the tuning of the models parameters. Fabrication imperfections not only during etching but also for wafer bonding may explain the perceived differences. Especially fluidic damping of the moving structures is quite sensitive to the curves. Following the peak heights the actuators resonance and the ear simulators resonance differ in both the SPL and THD measurements. As revealed in the THD measurement, the K3 fraction is dominating. Seemingly stochastic measurement variations for low-frequency THD values are attributed to limits of the microphone setup, since 1% translates to −40 dB in measuring value.

The demonstration of the linear bending actuators showed their application in a MEMS μSpeaker. The unique bending capabilities not only allowed relevant performance indicators to be significantly enhanced, but also could potentially allow for reduced device sizes. This is of major interest for the commercialization of any MEMS device. In this context, it is important to mention that the silicon fabrication technology is compatible with complementary metal-oxide semiconductor technology.

## Perspective

Further work will focus on technology development to decrease the minimum possible gap distances and increase area utilization at the same time. One major challenge is to keep the electrical capacitance low, not only for the actuators but also for sources of additional parasitic capacitances.

In the field of MEMS-based speakers, quite impressive piezoelectric speakers are at this moment in time probably the most advanced (e.g. USound). Other approaches (e.g. AudioPixels) have, to the best knowledge of the authors, so far had no or only very little experimental data relevant to practical applications published. When compared to accessible data, the μSpeakers presented in this paper have an attractive performance profile. The electrostatic actuation principle does not encounter a piezoelectric hysteresis or intrinsic power dissipation mechanisms^28^, resulting in a THD < 1% without any further acoustic design or signal preprocessing. The linearization strategies which furnish macroscopic electrostatic speakers with their spectacular performance have successfully been translated to the low-voltage MEMS world.

The electrostatic μSpeakers presented in this paper also promise major progress regarding power consumption and peak current draw. The batteries of advanced in-ear devices are tiny (typical 60 mAh). Most of the battery budget is reserved for smart functions such as speech recognition and wireless connectivity. This confines the power available to the audio reproduction system to only a small, single-digit number of milliwatts. MEMS μSpeakers must beat this target to be competitive with classical electrodynamic or balanced armature speakers. The relevant key parameter for the battery drain caused by the transducer is the electrical capacity. The capacity determines the reactive peak current and the reactive power that must be handled by the inevitably power-dissipating drive electronics. The μSpeakers presented here feature a total electric capacity of less than 1 nF. In comparison, capacity values in excess of 20 nF or even 150 nF have been published for piezoelectric systems (USound, xMEMS). The beneficial combination of a comparatively low signal voltage and a low actuator capacity allows the presented μSpeaker to be driven with small, fully integrated charge pumps connected to a small lithium polymer or zinc-air battery.

While a fair comparison should be between μSpeakers that are all able to deliver peak SPLs of say about 120 dB across the relevant parts of the audio spectrum, an assessment of the adiabatic work required to generate 120 dB in the ear channel in relation to the energy content of the electrical field available from the electrostatic cells reveals that it should be possible to design an electrostatic 120 dB MEMS μSpeaker with a capacitance far below 1nF.

## Materials and methods

### Finite element analysis

In the main paper body we gave the relevant principles, which lead to the constitution of the design of the actuator and specifically of the electrodes shape. Starting from a three-electrode basis having three straight electrodes, it is beneficial to have a suspension bridge arrangement because of its mechanical susceptibility to bending. Thus, the general shape is set. Parameters which need to be defined are: the length of the electrodes parallel to the longitudinal axis of the actuator, the electrodes distances or gap, their thicknesses and the height of the electrodes anchor. Due to symmetry reasons there are just two electrode thicknesses, i.e. middle and outer electrode, and one gap thickness to be defined. All these parameters mentioned before are dependent from each other. For example, a long electrode (e.g. >600 μm) would require a bigger gap (e.g. >4 μm) for a fixed voltage to not to be susceptible to pull-in. Conversely, longer electrodes are softer, thus enabling higher deflections with the same force. To address all these dependencies and include actual features of a possible fabrication design, finite element analysis seem suited to define the parameters.

As a method, we implemented the Covariance Matrix Adaptation Evolution Strategy^41^. A method that searches for beneficial sets of parameters by calculating several individuals and adapt the search over several generations with then newly created individuals. This method seems suitable because the behavior of the actuator based on the dependencies is noisy. In total, several thousands of individuals have been calculated.

We used a 2D model which implements both the mechanical and the electrostatic domain. Calculations were carried out using ANSYS Software and by implementing plane223 elements which are capable of both domains mentioned before. The list of results with the parameter sets and their respective maximum deflection was then sorted regarding this variable of merit. This parameter set was implemented in the layout process for fabrication. Table 1 shows the parameter set chosen for the layout.

**Table 1:** Parameter set chosen for the layout for fabrication

Parameter	Value	Unit
Electrode length	430	μm
Middle electrode thickness	5	μm
Outer electrode thickness	2	μm
Electrode distance/gap	2.5	μm
Height of electrode anchor	34	μm

### Lumped-parameter model

If a push-pull, spring-capacitor system can be used to describe the motion of the actuator, there will be a set of parameters (i.e. capacitance and spring constant) for which the S-curve obtained by finite-element simulations can be reproduced analytically. The electrostatic gap is set by design (2.5 μm), and the zero-voltage capacitance can be obtained by geometrical considerations. In order to describe the mechanical amplification achieved by the NED mechanism, we have introduced a lever factor *θ* that couples the electrode displacement and the motion of the tip, as was proposed by Spitz et al.^31^. This lever factor and the equivalent spring constant can be obtained by performing a least-squares fit using equation (11), inserting the simulated values of the deflection for the corresponding AC and DC voltages. The obtained parameters are shown in Table 2 and the comparison of the analytical curve and the finite-element simulation is shown in Fig. 8b. It can be confirmed that the analytical S-curve describes the motion of the actuator very well. Given that fabrication tolerances have an impact on the actual geometry of the device, these parameters were slightly adjusted when comparing them to the measurements of the speakers. The Q-factor was obtained by trial and error, in particular by comparing with the THD curve, which is very sensitive to this value.

11 $${k}_{eff}\left(\frac{w}{\theta }\right)=\frac{{C}_{0}}{2{g}_{0}}\left[\frac{{({u}_{dc}+{u}_{ac})}^{2}}{{(1-\frac{w}{\theta {g}_{0}})}^{2}}-\frac{{({u}_{dc}-{u}_{ac})}^{2}}{{(1+\frac{w}{\theta {g}_{0}})}^{2}}\right]$$

**Table 2:** Parameters used in the equivalent circuit model

Parameter	Fitting against FEM	Adjustment to experiments
Spring constant, *k*_*eff*_	97.8 N/m	77.8 N/m
NED lever factor, *θ*	10.8	9.81
Effective mass, *m*_*eff*_	1.25 × 10^−7^ kg	9.96 × 10^−8^ kg
Quality factor, *Q*	–	2.5

In order to simulate this lumped-parameter model as an equivalent circuit, the procedure proposed by Monsalve et al.^33^ was implemented for the case of the push-pull actuator. This work describes how the large-signal behavior of a spring-capacitor system can be fully represented by an equivalent circuit, which allows its harmonic distortion and the interaction with the acoustical load (IEC 60318-4 ear simulator) to be simulated. This equivalent circuit (Fig. 8a) is composed of cross-coupled loops that represent the electrical, mechanical and acoustic domains. The electrical domain (left-hand side) contains the two variable capacitors connected in series. The mechanical domain portrays the force balance (Eq. (11)), with the two opposing electrostatic forces. The acoustic load is found on the right-hand side, where air is pumped by the actuator through the slits (including some leakage) towards the ear simulator. This lumped-parameter model is able to the predict the behavior of the μSpeaker device over a broad range of frequencies.
